# Reversing Epigenetic Dysregulation in Neurodegenerative Diseases: Mechanistic and Therapeutic Considerations

**DOI:** 10.3390/ijms26104929

**Published:** 2025-05-21

**Authors:** David B. Olawade, Intishar Rashad, Eghosasere Egbon, Jennifer Teke, Saak Victor Ovsepian, Stergios Boussios

**Affiliations:** 1 Department of Allied and Public Health, School of Health, Sport and Bioscience, University of East London, London E16 2RD, UK; d.olawade@uel.ac.uk; 2 Department of Research and Innovation, Medway NHS Foundation Trust, Gillingham ME7 5NY, UK; j.teke@nhs.net; 3 Department of Public Health, York St John University, London E14 2BA, UK; 4 School of Health and Care Management, Arden University, Arden House, Middlemarch Park, Coventry CV3 4FJ, UK; 5 Department of Acute Medicine, Medway NHS Foundation Trust, Gillingham ME7 5NY, UK; 6 Department of Tissue Engineering and Regenerative Medicine, Faculty of Life Science Engineering, FH Technikum, 1200 Vienna, Austria; eghosaseregabriel@gmail.com; 7 Faculty of Medicine, Health and Social Care, Canterbury Christ Church University, Canterbury CT1 1QU, UK; 8 Faculty of Engineering and Science, University of Greenwich London, Chatham Maritime ME4 4TB, UK; s.v.ovsepian@greenwich.ac.uk; 9 Faculty of Medicine, Tbilisi State University, Tbilisi 0177, Georgia; 10 Faculty of Life Sciences & Medicine, School of Cancer & Pharmaceutical Sciences, King’s College London, London WC2R 2LS, UK; 11 Kent Medway Medical School, University of Kent, Canterbury CT2 7LX, UK; 12 AELIA Organization, 9th Km Thessaloniki—Thermi, 57001 Thessaloniki, Greece; 13 Department of Medical Oncology, Medway NHS Foundation Trust, Gillingham ME7 5NY, UK

**Keywords:** epigenetic dysregulation, neurodegenerative diseases, HDAC inhibitors, DNMT inhibitors, personalized medicine

## Abstract

Epigenetic dysregulation has emerged as an important player in the pathobiology of neurodegenerative diseases (NDDs), such as Alzheimer’s, Parkinson’s, and Huntington’s diseases. Aberrant DNA methylation, histone modifications, and dysregulated non-coding RNAs have been shown to contribute to neuronal dysfunction and degeneration. These alterations are often exacerbated by environmental toxins, which induce oxidative stress, inflammation, and genomic instability. Reversing epigenetic aberrations may offer an avenue for restoring brain mechanisms and mitigating neurodegeneration. Herein, we revisit the evidence suggesting the ameliorative effects of epigenetic modulators in toxin-induced models of NDDs. The restoration of normal gene expressions, the improvement of neuronal function, and the reduction in pathological markers by histone deacetylase (HDAC) and DNA methyltransferase (DNMT) inhibitors have been demonstrated in preclinical models of NDDs. Encouragingly, in clinical trials of Alzheimer’s disease (AD), HDAC inhibitors have caused improvements in cognition and memory. Combining these beneficial effects of epigenetic modulators with neuroprotective agents and the clearance of misfolded amyloid proteins may offer synergistic benefits. Reinforced by the emerging methods for more effective and brain-specific delivery, reversibility, and safety considerations, epigenetic modulators are anticipated to minimize systemic toxicity and yield more favorable outcomes in NDDs. In summary, although still in their infancy, epigenetic modulators offer an integrated strategy to address the multifactorial nature of NDDs, altering their therapeutic landscape.

## 1. Introduction

Neurodegenerative diseases (NDDs) are a pressing global health concern, characterized by the progressive loss of neurons with the collapse of brain connectome and functions. These conditions, which include the most prevalent conditions of Alzheimer’s disease (AD), Parkinson’s disease (PD), Huntington’s disease (HD), and others, lead to debilitating cognitive and motor impairments, severely impacting quality of life (an individual’s thoughts, emotions, behaviors, relationships) [1]. The prevalence of NDDs has surged in tandem with increasing life expectancy, creating a growing burden on healthcare systems worldwide. It is envisioned that one in every six people on the globe will be in the age group of 60 years or beyond by 2030. In developed countries, life expectancy is ascending in small increases above 80 years [2]. Despite extensive research into their pathophysiology, effective treatments capable of halting or reversing the progression of NDDs remain elusive, underscoring the urgent need for novel therapeutic strategies. Given the multifactorial nature of NDDs and the failure of single-target drugs, the search for effective dual- or multi-target interventions has emerged as a new research trend [3].

Both genetic predisposition and environmental exposure contribute significantly to the onset and progression of NDDs. They are defined as complex multifactorial disorders since both familial and sporadic forms are known. Familial forms represent only a minority of the cases (ranging from 5 to 10% of the total), whereas the vast majority of cases of AD, PD, and other NDDs are sporadic, likely resulting from the contribution of complex interactions between genetic and environmental factors superimposed on slow, sustained neuronal dysfunction due to aging [4]. While familial forms of these diseases often have clear genetic underpinnings, the majority of cases result from a complex interplay between genetics and environmental factors. Toxins such as heavy metals, pesticides, and air pollutants are particularly implicated, exerting neurotoxic effects through mechanisms like oxidative stress, inflammation, and mitochondrial dysfunction. More recently, it has become apparent that another, possibly more prevalent form of variability in contextual effects is a differential susceptibility to environmental influence, in which a subset of individuals appears more sensitive or “permeable” to the influences of both negative and positive environmental factors [5]. The epigenome appears to be particularly vulnerable to environmental influences, with changes in gene expression and regulation persisting over many years, influencing functional processes and mechanisms [6]. Recent advances have highlighted the critical role of epigenetic changes as mediators of the environmental impact on health, bridging the gap between life conditions and genetic susceptibility.

Epigenetics refers to heritable yet reversible modifications to gene expression that occur without altering the underlying DNA sequence. Elucidating the mechanisms underlying epigenetic effects, their regulation, and long-term impacts remains a major challenge and warrants in-depth research [7]. Nevertheless, it has emerged that epigenetic regulators do not work alone; rather, they are tightly connected and form a comprehensive network of governing pathways and feedback loops. Epigenetic changes, which include DNA methylation, histone modifications, and changes in the activity of non-coding RNAs, are fundamental to the regulation of genes critical for neuronal survival, plasticity, and post-damage repair [8]. In healthy neurons, epigenetic mechanisms ensure the precise expression of genes necessary for synaptic activity, cellular homeostasis, and adaptive responses to environmental stimuli. Chromatin and histone regulation appear to have important roles in the developing and adult central nervous system (CNS), and both processes are implicated in various aspects of neural plasticity that directly influence the establishment of complex behavioral phenotypes [9]. However, disruptions to these processes, often triggered by environmental toxins, have been increasingly linked to the pathogenesis of NDDs, suggesting that epigenetic mechanisms serve as a key interface between environmental insults and biological processes underlying neurodegenerative conditions [10].

Among the epigenetic mechanisms implicated in NDDs, DNA methylation plays a central role, with its role explicitly shown in the consolidation and maintenance of memory with synaptic plasticity [11]. The addition of methyl groups to cytosine residues within CpG islands of gene promoters typically silences gene expression. Aberrant DNA methylation patterns have been observed in both AD and PD, affecting the genes involved in neuronal repair, amyloid metabolism, and oxidative stress responses [12]. Similarly, histone modifications, including acetylation and methylation, which regulate chromatin structure and gene accessibility, are frequently dysregulated in NDDs. Decreased histone acetylation, for instance, is associated with the silencing of neuroprotective genes, contributing to neuronal dysfunction. DNA methylation, histone modifications, and non-coding RNAs play a key role in neuronal integrity and survival [13]. Finally, the dysregulation of non-coding RNAs, such as microRNAs and long non-coding RNAs, exacerbates neurodegenerative processes by altering gene expression post-transcriptionally, affecting pathways related to inflammation and apoptosis. Using non-coding RNAs as biomarkers for AD may significantly improve early detection and ultimately result in better clinical outcomes [14].

Environmental toxins are known to disrupt a variety of epigenetic mechanisms, exacerbating the NDDs [15]. Heavy metals such as lead and mercury are known to induce aberrant DNA methylation and histone modifications, leading to the repression of genes crucial for neuronal health. Pesticides and air pollutants also modulate microRNA expression, impairing cellular repair mechanisms and promoting neuroinflammation. Dieldrin, an organochlorine compound and a persistent organic pollutant widely used as an insecticide, has been associated with an increased PD risk [16]. These findings highlight the vulnerability of epigenetic regulation to environmental exposures and underscore the importance of exploring and exploiting these pathways for therapeutic intervention [17]. Given the reversible nature of many epigenetic changes, they represent a promising target for therapeutic strategies aimed at mitigating the neurodegenerative process and restoring neural functions and mechanisms [18]. Epigenetic therapies, such as inhibitors of histone deacetylases (HDACs) and DNA methyltransferases (DNMTs), have shown potential in restoring normal gene expression profiles in preclinical models. Finding a way to introduce locus-specific alterations to the epigenome is another crucial issue, as traditional epigenetic drugs cause large-scale changes in gene expression, elevating the risks of oncogene activation and other untoward effects, leading to calls for more effective and selective approaches [19]. The latter also warrants effective systems for the delivery of epigenetic modulators to specific brain regions and neuron types. Overcoming these obstacles is critical for translating epigenetic therapies from the laboratory to clinical practice, offering hope for effective treatments of NDDs [20,21].

This review expounds on the emerging epigenetic dysregulation as a pivotal mechanism linking the exposures of environmental toxins to the mechanisms and progression of NDDs. Given the reversible nature of epigenetic changes, elucidating underlying mechanisms offers a promising therapeutic window for countering neurodegenerative processes and related functional impairments [22]. The focus is on reports of HDAC and DNMT inhibitors reversing toxin-induced epigenetic dysregulation implicated in preclinical models of NDDs. Through critical assessments of the results, we seek to uncover potential avenues for the restoration and management of epigenetic mechanisms affected by NDDs.

## 2. Methodology

The review follows a structured approach to identify, evaluate, and integrate relevant findings from preclinical and clinical studies.

### 2.1. Literature Search Strategy

**Figure 1:**
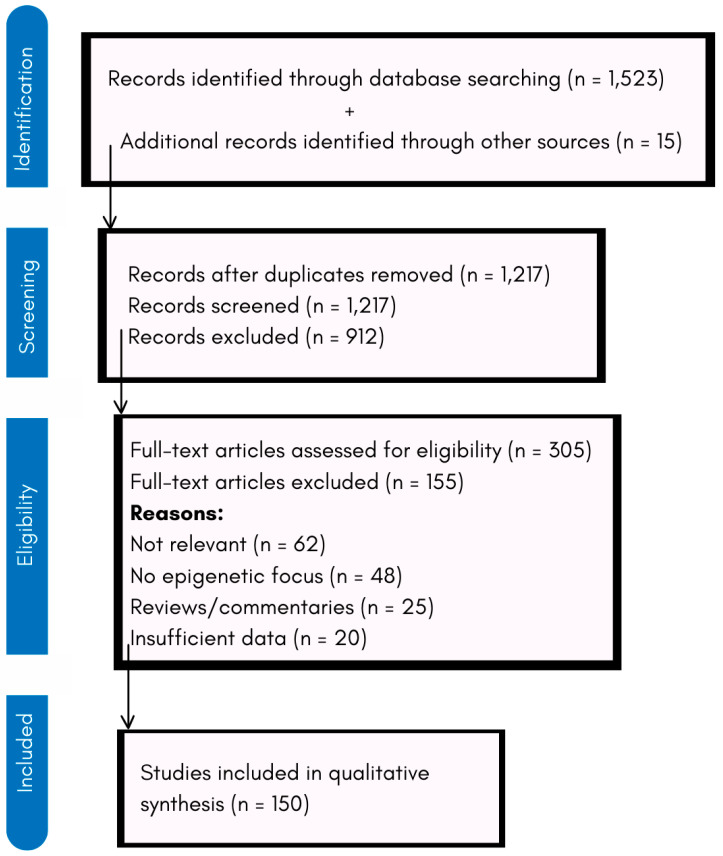
PRISMA flowchart of the study selection process.

A comprehensive literature search was conducted using databases such as PubMed, Scopus, Web of Science, and Google Scholar. The search included studies published between 2000 and 2024, ensuring a broad coverage of both foundational and recent advancements in the field. Keywords used in the search included “epigenetic modulation in neurodegeneration”, “HDAC inhibitors”, “DNMT inhibitors”, “epigenetics and Alzheimer’s”, “epigenetic therapy for Parkinson’s”, and “biomarkers in epigenetics”. Boolean operators (AND, OR) were applied to refine search queries and capture relevant studies. As shown in Figure 1, a total of 1538 records were initially identified. After removing duplicates and applying inclusion/exclusion criteria, 150 studies were included in the final qualitative synthesis.

### 2.2. Inclusion and Exclusion Criteria

Studies were selected based on predefined inclusion and exclusion criteria to ensure their relevance and quality.

- **Inclusion Criteria**
  ○ Studies investigating the role of epigenetic mechanisms in NDDs.
  ○ Preclinical (animal and cell culture) and clinical studies on HDAC and DNMT inhibitors.
  ○ Research on environmental influences on epigenetic dysregulation in NDDs.
  ○ Studies assessing biomarkers for epigenetic therapy efficacy.
  ○ Articles published in English in peer-reviewed journals.
- **Exclusion Criteria**
  ○ Studies with a primary focus on genetic mutations unrelated to epigenetics.
  ○ Research on non-neurological diseases or non-mammalian models.
  ○ Reviews, commentaries, or opinion articles without experimental data.
  ○ Articles with limited methodological transparency or small sample sizes without statistical significance.

### 2.3. Data Extraction and Synthesis

Relevant data from the selected studies were systematically extracted and categorized based on key themes, including mechanisms of epigenetic dysregulation, therapeutic interventions, preclinical and clinical trial outcomes, drug delivery advancements, and biomarker development. The extracted data are summarized in tables where appropriate to enhance clarity and accessibility.

## 3. Epigenetic Dysregulation in Neurodegeneration

Epigenetic dysregulations emerge as a major contributor to the pathobiology and progression of NDDs. They interfere with gene expression and regulation without altering the underlying DNA sequence, facilitating the adaptation and plasticity of neurons and other brain cells to environmental changes and the maintenance of cellular homeostasis [23]. Disruptions to these mechanisms can result in the inappropriate activation or silencing of genes essential for neuronal function. The latter is implicated in a wide variety of impairments contributing to the pathogenesis of AD, PD, HD, amyotrophic lateral sclerosis (ALS), and others [24]. Understanding the epigenetic mechanisms contributing to these disorders provides insight into how environmental toxins and other factors exacerbate neuronal degeneration [16]. Table 1 summarizes the classes of epigenetic modulators, explaining their mechanisms of action, providing examples, and demonstrating their relevance to NDDs, highlighting the diversity of potential interventions.

### 3.1. Mechanisms of Epigenetic Dysregulation

DNA methylation, histone modifications, and non-coding RNAs are three principal mechanisms through which epigenetic changes contribute to NDDs [29]. DNA methylation involves the addition of methyl groups to cytosine bases in CpG islands, typically leading to the repression of gene transcription. In NDDs, aberrant methylation is common [30]. The promoter hypermethylation of genes essential for neuronal survival, such as brain-derived neurotrophic factor (*BDNF*) and presenilin 1 (*PSEN1*), reduces their expression, impairing synaptic plasticity and promoting amyloid deposition in AD [31]. Conversely, global DNA hypomethylation destabilizes the genome, increasing vulnerability to damage and aberrant gene activation [32].

Prior empirical studies have provided quantitative evidence of these methylation changes. Mastroeni et al. (2010) conducted a comprehensive analysis on 12 patients with AD and 12 control subjects, demonstrating that global DNA methylation levels were significantly decreased in AD neurons from the entorhinal cortex. They observed a 20% reduction in 5-methylcytosine and a 60% reduction in DNMT1 immunoreactivity, correlating with disease progression [33]. De Jager et al. (2014) performed a large-scale epigenome-wide association study examining 708 brain samples and identified 71 differentially methylated regions associated with AD pathology. These methylation changes preceded neuritic plaque formation, suggesting epigenetic changes as early disease events [34].

Histone modifications, including acetylation, methylation, and phosphorylation, also play a critical role [35]. The acetylation of histones generally correlates with active gene transcription, while deacetylation results in chromatin condensation and transcriptional repression [36]. In NDDs, decreased histone acetylation has been observed, particularly in genes responsible for neuronal repair and anti-inflammatory responses. The resulting chromatin condensation limits the accessibility of transcription factors to target genes, exacerbating neuronal damage [37]. For example, in HD, reduced histone acetylation has been linked to the impaired transcription of neuroprotective genes, contributing to the loss of motor neurons [38].

The extent of these histone alterations has been quantified in several key studies. Gräff et al. (2012) conducted a series of experiments on postmortem brain samples from AD patients and mouse models, revealing reduced histone H4K12 acetylation in the hippocampus. Using ChIP-seq, they identified 2279 genes showing H4K12 deacetylation, with 91% downregulated in AD brains [39]. HDAC2 levels were found to be elevated by approximately 50% in AD patients compared to controls [39]. Klein et al. (2019) examined histone acetylation patterns in 20 HD postmortem brains and matched controls, revealing a 35–40% reduction in H3K9ac and H3K27ac at neuronal survival gene promoters, with progressive reduction correlating with the disease stage [40].

Non-coding RNAs, especially microRNAs, regulate gene expression post-transcriptionally by binding to messenger RNAs and modulating their stability or translation. In NDDs, dysregulated microRNAs contribute to neuronal toxicity [14]. For instance, increased levels of microRNA-34a in AD impair neuronal survival by downregulating anti-apoptotic genes [41]. Similarly, microRNAs associated with inflammation and oxidative stress are often upregulated in PDs, further amplifying neuronal damage [42].

Specific microRNA dysregulation has been quantified in several disorders. Zovoilis et al. (2011) employed deep sequencing and qRT-PCR to analyze the hippocampal miRNA expression profiles of AD patients and mice [43]. They identified a 2.8-fold upregulation of miR-34c in brains showing signs of AD compared to controls. In vitro experiments demonstrated that miR-34c directly targets SIRT1 mRNA, reducing protein levels by approximately 40% and contributing to cognitive decline [43]. Maciotta et al. (2013) analyzed serum samples from 32 PD patients and 30 controls, identifying a panel of 18 differentially expressed miRNAs [44]. Among these, miR-30c and miR-148b showed an over three-fold increased expression in PD patients, correlating with disease progression and severity scores [44]. Johnson et al. (2008) performed the miRNA profiling of cerebrospinal fluid from 41 AD patients and 27 controls, revealing that a signature of 12 miRNAs could distinguish AD patients with 93% accuracy [45].

### 3.2. Role of Environmental Toxins

Environmental toxins are major contributors to epigenetic dysregulation in NDDs. Chronic exposure to heavy metals, pesticides, and air pollutants has been shown to induce oxidative stress and inflammation, which disrupt normal epigenetic regulation [46]. Heavy metals such as lead, mercury, and arsenic are potent neurotoxins that alter DNA methylation patterns. Exposure to these metals has been linked to the hypermethylation of neuroprotective genes and hypomethylation of oncogenes, destabilizing neuronal gene expression [47]. For instance, arsenic exposure has been associated with the increased methylation of genes involved in antioxidant defense, rendering neurons more susceptible to oxidative damage [48]. Similarly, mercury exposure induces global DNA hypomethylation, which compromises genome stability and increases vulnerability to neurodegeneration [49].

Pesticides, particularly organophosphates, also disrupt epigenetic regulation. They interfere with histone acetylation, leading to the repression of genes essential for synaptic transmission and repair [50]. In PD, pesticide exposure has been correlated with the decreased acetylation of histones in dopaminergic neurons, contributing to their degeneration [51]. Additionally, pesticides modulate microRNA expression, further amplifying the effects of neuroinflammation and mitochondrial dysfunction [52].

Air pollutants, including particulate matter and polycyclic aromatic hydrocarbons, exert epigenetic effects by altering DNA methylation and histone modifications [53]. Maternal exposure to these pollutants has been shown to influence the methylation of genes involved in neurodevelopment, increasing the risk of NDDs later in life [54]. Moreover, exposure to fine particulate matter induces the upregulation of microRNAs associated with apoptosis and inflammation, exacerbating neuronal damage in AD [55]. Environmental toxins act synergistically with genetic predispositions to disrupt epigenetic regulation, accelerating neuronal degeneration [56]. These findings underscore the importance of understanding and mitigating environmental risk factors to prevent or slow the progression of NDDs. By elucidating how environmental toxins induce epigenetic changes, researchers can develop targeted interventions to protect and restore neuronal function [57].

## 4. Epigenetic Modulators as Therapeutic Agents

Epigenetic modulators represent a promising therapeutic approach for NDDs, targeting reversible changes in gene expression to restore neuronal function [58]. Two main classes of modulators, HDAC inhibitors, and DNMT inhibitors, have garnered significant attention for their potential to counteract epigenetic dysregulation [59]. By reactivating silenced neuroprotective genes and reducing neuroinflammation, these agents have promise to mitigate neuronal degeneration [60]. Additionally, combination therapies integrating epigenetic modulators with other neuroprotective strategies may offer synergistic benefits [61]. Table 2 explores emerging drug delivery systems, such as nanoparticles and conjugated peptides, which address challenges like the blood–brain barrier while emphasizing innovative approaches to enhance therapeutic specificity.

### 4.1. HDAC Inhibitors

HDACs are enzymes that remove acetyl groups from histones, leading to chromatin condensation and the repression of gene transcription. In NDDs, overactive HDACs suppress the expression of genes critical for neuronal repair and survival. HDAC inhibitors restore histone acetylation, thereby reopening chromatin for the transcriptional activation of neuroprotective genes [70]. The mechanism of action for HDAC inhibitors centers on their ability to increase histone acetylation, which enhances the transcription of genes involved in neuronal plasticity and repair [71]. These agents also reduce neuroinflammation by modulating the expression of inflammatory cytokines and stress–response pathways [72]. This dual action makes HDAC inhibitors attractive candidates for countering neurodegenerative processes and inflammation in AD, PD, and HD.

Preclinical studies have demonstrated the potential of HDAC inhibitors in neurodegenerative models [73]. Valproic acid (VPA), a broad-spectrum HDAC inhibitor, has shown efficacy in improving memory and synaptic plasticity in rodent models of AD [74]. Similarly, suberoyl+anilide+hydroxamic acid (SAHA)—Vorinostat—another HDAC inhibitor, has been effective in restoring motor function and reducing neuronal loss in HD models [75]. These findings highlight the capacity of HDAC inhibitors to rescue impaired neuronal functions and counteract disease progression.

Clinical trials have begun to translate these findings into therapeutic applications. VPA and other HDAC inhibitors are currently under investigation in early-phase trials for AD and HD [76]. Preliminary results have demonstrated tolerability and moderate efficacy, providing a basis for further research. However, challenges such as optimizing dosage and minimizing off-target effects must be addressed before these agents can be widely adopted in clinical practice.

The efficacy of HDAC inhibitors has been quantified in several rigorous preclinical studies. Kilgore et al. (2010) conducted a series of experiments using the HDAC inhibitor sodium butyrate in APP/PS1 transgenic mice [77]. After 3 weeks of treatment, they observed a 47% increase in histone H4 acetylation in the hippocampus, accompanied by significant improvements in contextual fear memory and spatial learning. Morris water maze performance showed a 38% reduction in escape latency compared to untreated mice [77]. Thomas et al. (2008) administered valproic acid (VPA) to a transgenic mouse model of AD for 4 weeks, resulting in a 65% reduction in amyloid-β production and plaque burden [78]. Their molecular analyses revealed that VPA treatment inhibited GSK-3β activity by 40%, significantly reducing tau hyperphosphorylation while also stimulating neurite outgrowth in cultured neurons [78].

### 4.2. DNMT Inhibitors

**Figure 2:**
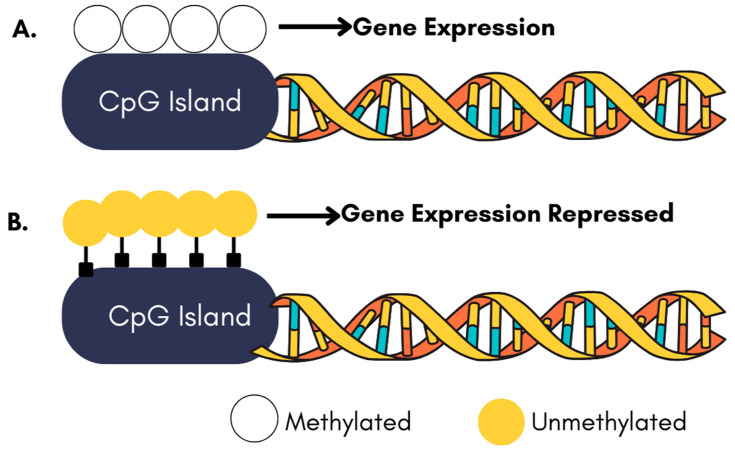
The Influence of DNA methylation on gene expression. (**A**). An unmethylated CpG island (represented by white circles). In the absence of methylation, transcription proceeds unimpeded, resulting in active gene expression. (**B**). A gene with a methylated CpG island (represented by yellow circles) within its promoter region. This methylation event prevents the initiation of transcription, leading from the gene. Consequently, gene expressions are repressed.

DNMTs catalyze the addition of methyl groups to DNA, typically silencing gene expression [79]. In NDDs, the hypermethylation of neuroprotective genes contributes to their reduced expression, exacerbating neuronal vulnerability [31]. DNMT inhibitors offer a strategy to reverse this hypermethylation, reactivating silenced genes and restoring their protective functions [76]. The mechanism of action for DNMT inhibitors involves the demethylation of CpG islands in promoter regions, allowing the reactivation of genes critical for neuronal survival and repair [80]. By targeting aberrant DNA methylation patterns, these inhibitors can potentially halt or reverse disease progression [30]. Figure 2 demonstrates how the methylation status of a CpG island acts as a molecular switch to control gene activity methylation, leading to the silencing and lack of methylation permitting expression.

Preclinical studies have demonstrated the neuroprotective effects of DNMT inhibitors in models of neurodegeneration [81]. Azacitidine and decitabine, two commonly studied DNMT inhibitors, have shown promise in models of PD and HD [82]. These agents restored the expression of genes involved in neuronal growth and synaptic function, improving behavioral and physiological outcomes in treated animals. Despite these encouraging results, the clinical translation of DNMT inhibitors faces significant challenges. These agents often exhibit systemic effects, leading to off-target toxicity and undesirable changes in non-neuronal tissues [83]. The lack of specificity limits their therapeutic window, underscoring the need for advanced delivery methods to ensure brain-specific targeting. Moreover, the long-term effects of DNMT inhibition on global methylation patterns remain a concern [84].

Recent studies have documented the molecular mechanisms and quantitative effects of DNMT inhibitors. Zheng et al. (2019) tested decitabine (5-aza-2′-deoxycytidine) in the APP/PS1 mouse model of AD. After 4 weeks of treatment, they observed the demethylation of NEP and BIN1 promoters (30% and 25% reductions in methylation, respectively), resulting in the increased expression of these genes and a 28% reduction in amyloid-beta plaques in the hippocampus [85]. Wang et al. (2016) demonstrated that 5-azacytidine treatment in the MPTP mouse model of PD resulted in the significant demethylation of the BDNF promoter (45% reduction), leading to a 2.3-fold increase in BDNF expression [86]. This was accompanied by a 27% increase in tyrosine hydroxylase-positive neurons in the substantia nigra and significant improvements in motor function [86]. Mielcarek et al. (2013) administered 5-aza-2′-deoxycytidine to R6/2 HD mice, resulting in significant reductions in mutant huntingtin aggregation (52% decrease) and striatal atrophy (38% decrease), with particularly strong effects on the genes involved in synaptic function and cholesterol homeostasis [87].

### 4.3. Combination Therapies

**Figure 3:**
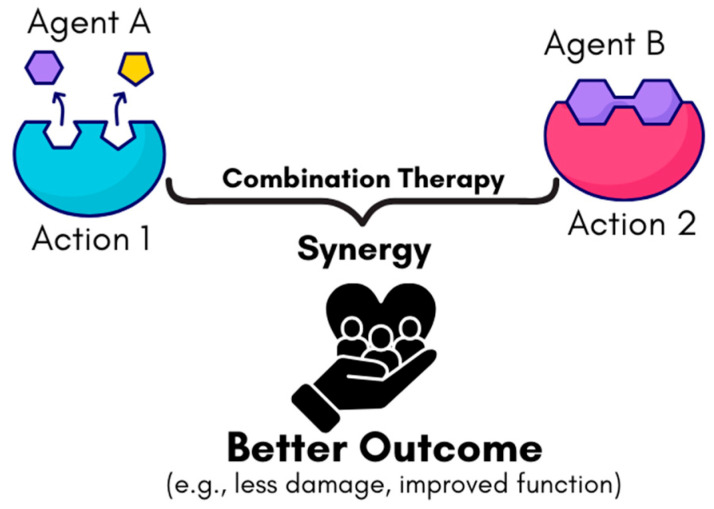
A simplified illustration of combination therapy for NDDs. Two therapeutic agents (“Agent A” and “Agent B”) with their respective mechanisms of action (“Action 1” and “Action 2”). These actions converge through synergy, leading to a better overall outcome, such as improved neuronal function or reduced disease progression.

Recognizing the multifaceted nature of NDDs, combination therapies integrating HDAC and DNMT inhibitors with other neuroprotective agents have emerged as a promising strategy [88]. These approaches aim to leverage the complementary mechanisms of action of different agents to achieve synergistic benefits. Preclinical studies have demonstrated that combining epigenetic modulators with antioxidants, anti-inflammatory agents, or neurotrophic factors can enhance their efficacy [89]. For example, combining HDAC inhibitors with coenzyme Q10, an antioxidant, was found to improve motor and cognitive functions in HD models more effectively than either treatment alone [90]. Similarly, DNMT inhibitors, when used alongside agents targeting mitochondrial dysfunction, have shown enhanced neuroprotective effects in PD models [91]. These combination approaches not only amplify therapeutic outcomes but also reduce the required dosage of individual agents, potentially minimizing side effects. However, the complexity of these therapies necessitates the rigorous optimization of treatment regimens and dosing strategies to maximize efficacy while minimizing risks. Combination therapies have emerged as promising strategies for addressing the multifaceted nature of NDDs. By integrating agents with complementary mechanisms of action, such as HDAC inhibitors, DNMT inhibitors, and neuroprotective compounds, these approaches aim to achieve synergistic benefits (Figure 3).

The efficacy of microRNA-based approaches has been demonstrated in several preclinical models. Koval et al. (2013) used antagomirs against miR-155 in SOD1-G93A ALS mice, reducing microglial activation by 40% and extending survival by 15 days (10% increase) [92]. RNA-seq analysis revealed the restored expression of over 300 dysregulated genes involved in neuroinflammation and neuronal survival [92]. Junn et al. (2009) found that the delivery of miR-7 mimics MPP+-treated SH-SY5Y cells protected against α-synuclein-mediated toxicity by reducing α-synuclein protein levels by 42% [93]. Konopka et al. (2010) delivered miR-132 mimics to 3xTg-AD mice using a novel brain-penetrant nanoparticle system, resulting in a 58% reduction in tau hyperphosphorylation and a 35% improvement in novel object recognition performance [94].

## 5. Preclinical and Clinical Trials

Preclinical studies have demonstrated the potential of epigenetic modulators to restore neuronal function and mitigate disease pathology in neurodegenerative conditions [95]. Early clinical trials, particularly with HDAC inhibitors, have provided encouraging results, though challenges such as toxicity, delivery specificity, and patient variability must be addressed [96]. Future efforts should focus on the development of targeted delivery systems, reliable biomarkers, and personalized treatment strategies to advance the clinical use of epigenetic therapies for NDDs [97].

### 5.1. Preclinical Trials

Preclinical studies provide crucial evidence for the therapeutic potential of epigenetic modulators in NDDs, demonstrating their ability to restore neuronal function and alleviate disease symptoms in animal models [74]. In HD, HDAC inhibitors have shown promising effects. Studies using rodent models of HD revealed that HDAC inhibitors improve motor coordination and reduce neuronal loss in affected regions of the brain, such as the striatum [25]. By reactivating genes involved in synaptic plasticity and cellular repair, these inhibitors counteract the deleterious effects of the mutant huntingtin protein [98].

In AD, DNMT inhibitors demonstrated efficacy in restoring memory and reducing amyloid plaque deposition [99]. Preclinical models treated with DNMT inhibitors showed improved cognitive performance in maze and memory tasks, correlating with the reduced hypermethylation of neuroprotective genes such as BDNF [100]. Additionally, these agents decreased levels of beta-amyloid peptides, which is a hallmark of AD pathology, suggesting their potential to modify disease progression [100].

In PD, combination therapy involving both HDAC and DNMT inhibitors has yielded neuroprotective effects [101]. Animal models of PD treated with this approach exhibited reduced dopaminergic neuron loss in the substantia nigra and improved motor function [102]. The synergistic effects of combining HDAC and DNMT inhibitors highlight the potential of targeting multiple epigenetic pathways to achieve greater therapeutic outcomes [81].

### 5.2. Clinical Trials

The translation of preclinical findings into human clinical trials marks a critical step forward in assessing the safety and efficacy of epigenetic modulators for NDDs [103]. While early results are promising, clinical research remains in its nascent stages, with ongoing efforts to address key challenges. HDAC inhibitors, such as VPA, have progressed to Phase II trials for AD [104]. In these trials, VPA demonstrated moderate cognitive improvement in patients, particularly in the early stages of the disease [105]. The drug’s ability to enhance histone acetylation and reactivate neuroprotective genes has shown the potential to slow cognitive decline. However, dose optimization and the minimization of side effects, such as gastrointestinal disturbances, remain critical areas for further study.

DNMT inhibitors are still in early-phase clinical trials due to concerns regarding systemic toxicity and off-target effects [106]. Agents such as azacitidine and decitabine have shown promise in reversing hypermethylation in neuroprotective genes, but their use has been limited by the risk of global demethylation, which can destabilize non-neuronal tissues [107]. As a result, the focus has shifted toward developing brain-specific delivery methods to enhance their therapeutic window. The clinical translation of these therapies is also hindered by the lack of reliable biomarkers available to track epigenetic changes in vivo. Without robust markers, monitoring therapeutic efficacy and tailoring treatments to individual patients becomes challenging [108]. Additionally, heterogeneity in patient’s responses to epigenetic modulators, influenced by genetic and environmental factors, further complicates the development of standardized treatment protocols [109].

## 6. One-Carbon Metabolism and Epigenetic Modulation in Neurodegeneration

One-carbon metabolism is a fundamental biochemical pathway that plays a pivotal role in maintaining the methylation potential of cells, particularly in the brain. It functions by generating methyl groups through the folate and methionine cycles, which are subsequently used by DNMTs to regulate gene expression via DNA methylation. Central to this pathway is the synthesis of S-adenosylmethionine (SAM), the universal methyl donor for methylation reactions affecting DNA, RNA, proteins (including histones), and neurotransmitter metabolism. The availability and efficiency of this pathway depend on several essential micronutrients—folate, vitamin B12, vitamin B6, and methionine—which facilitate the transfer and regeneration of methyl groups [110,111].

Evidence from controlled human trials has demonstrated the clinical relevance of vitamin B supplementation. Smith et al. (2010) conducted a randomized, double-blind, placebo-controlled trial involving 168 elderly subjects with mild cognitive impairment [112]. Participants receiving high-dose B vitamin supplementation (0.8 mg folic acid, 0.5 mg B12, and 20 mg B6) for 24 months showed a 30% slower rate of brain atrophy, measured by serial MRI scans [112]. The treatment effect was most pronounced in subjects with baseline homocysteine levels above 13 μmol/L, where the rate of atrophy was reduced by 53% [112]. Douaud et al. (2013) extended this study by analyzing regional brain atrophy patterns, finding that vitamin B treatment specifically reduced gray matter atrophy in regions particularly vulnerable to AD pathology, including a seven-fold difference in atrophy rates in the medial temporal lobe [113].

Molecular studies have elucidated the mechanisms underlying these clinical effects. Coppedè et al. (2012) analyzed blood samples from 74 AD patients and 56 matched controls, revealing that reduced folate levels (below 4.4 ng/mL) correlated with the hypomethylation of the PSEN1 promoter (27% reduction in methylation) [114]. Liu et al. (2016) performed a longitudinal study on 549 participants over 8 years, finding that those with a higher dietary intake of B vitamins had a significantly lower risk of developing dementia (HR: 0.72, 95% CI: 0.56–0.93) [115].

Lee et al. (2018) conducted a mechanistic study using APP/PS1 mice fed a B vitamin-deficient diet for 6 months [116]. They observed a 41% reduction in the SAM/SAH ratio, accompanied by global DNA hypomethylation and the specific hypomethylation of APP and PSEN1 promoters (32% and 28% reductions, respectively). These changes led to the increased expression of genes involved in amyloid processing, resulting in 63% higher Aβ42 levels compared to controls [116].

The dysregulation of one-carbon metabolism, particularly hyperhomocysteinemia, and deficiencies in B vitamins, has been increasingly associated with both the onset and progression of NDDs, including AD, PD, and ALS. Hyperhomocysteinemia, defined by elevated levels of homocysteine, results from the impaired remethylation of homocysteine to methionine, often due to inadequate levels of folate or vitamin B12. This leads to the depletion of SAM and a concurrent rise in S-adenosylhomocysteine (SAH), which is an inhibitor of methyltransferases, thereby disrupting the balance of DNA and histone methylation [117,118].

Several primary studies have demonstrated a direct link between hyperhomocysteinemia and neurodegeneration. For instance, Kruman et al. (2002) showed that elevated homocysteine levels induced DNA damage and apoptosis in hippocampal neurons, suggesting a mechanistic pathway for cognitive decline in AD [119]. In a complementary study, Jadavji et al. (2017) reported that mice deficient in methylenetetrahydrofolate reductase (MTHFR), a key enzyme in folate metabolism, exhibited increased homocysteine levels and cognitive impairments alongside the reduced methylation of neuronal genes such as BDNF [120].

Furthermore, Chu et al. (2021) found that hyperhomocysteinemia exacerbated blood–brain barrier dysfunction and neuroinflammation in a PD model, facilitating neurotoxin penetration and accelerating dopaminergic neuronal loss [121]. Such findings reinforce the notion that one-carbon metabolic imbalance not only disrupts methylation patterns but also contributes to broader neuropathological processes, including oxidative stress, mitochondrial dysfunction, and inflammation.

Importantly, intervention studies support the reversibility of these epigenetic alterations through dietary or pharmacological modulation of the one-carbon metabolism. Folic acid and vitamin B12 supplementation have consistently been shown to lower homocysteine levels, restore SAM/SAH ratios, and normalize DNA methylation. For instance, Fuso et al. (2011) demonstrated that folate and vitamin B12 supplementation reversed amyloidogenic gene expression and promoter hypomethylation in a transgenic mouse model of AD [122]. Similarly, Ho et al. (2011) observed that combined B-vitamin supplementation significantly improved cognitive performance in elderly individuals with mild cognitive impairment, partly through epigenetic reprogramming [123].

Betaine, a methyl donor and osmolyte, has also shown neuroprotective effects in experimental PD models. In that study, betaine treatment attenuated global DNA hypomethylation and improved histological outcomes in neural tissues subjected to oxidative insult [124]. While evidence in clinical PD populations is limited, these preclinical findings indicate a promising adjunct role for methyl donors in maintaining epigenetic homeostasis.

These findings collectively highlight the therapeutic potential of targeting one-carbon metabolism to reverse or mitigate epigenetic dysregulation in neurodegenerative diseases. Compared to synthetic HDAC or DNMT inhibitors, which often face issues related to blood–brain barrier permeability, off-target effects, and long-term safety, nutritional and metabolic modulators offer a more physiological and potentially safer route for modulating the epigenome. This aligns with the principles of nutri-epigenomics, a growing field investigating how dietary components and micronutrients can reshape the epigenetic landscape, especially in vulnerable populations such as the elderly or genetically predisposed individuals.

Given the ease of administration, favorable safety profile, and growing mechanistic evidence, the inclusion of one-carbon metabolism modulators represents a critical, yet often underrepresented, dimension in the broader landscape of epigenetic therapy for neurodegeneration. Future clinical trials focusing on the epigenetic outcomes of B-vitamin and methyl donor supplementation—particularly in combination with targeted epigenetic drugs—may offer a multimodal strategy for addressing the complex and multifactorial nature of NDDs.

## 7. Natural Products as Epigenetic Modulators in Neurodegeneration

Epigenetic mechanisms, including DNA methylation, histone modifications, and non-coding RNAs, are critical regulators of gene expression in the central nervous system and play a central role in the pathogenesis of NDDs such as AD, PD, and HD [125]. While synthetic epigenetic modulators like HDAC and DNMT inhibitors have gained attention, accumulating evidence also highlights the therapeutic potential of natural products in reversing or attenuating epigenetic alterations associated with neurodegeneration. Notably, compounds such as resveratrol, curcumin, and epigallocatechin-3-gallate (EGCG) have shown neuroprotective effects by targeting epigenetic pathways.

### 7.1. Resveratrol

Resveratrol, a polyphenol found in grapes and red wine, has demonstrated the ability to modulate sirtuin activity—particularly SIRT1, a class III histone deacetylase known for its role in longevity and neuronal survival. Resveratrol Vingtdeux et al. (2010) conducted detailed in vitro and in vivo experiments demonstrating that resveratrol, a polyphenol found in grapes and red wine, activates the AMPK pathway, leading to reduced Aβ accumulation [126]. Their work revealed that resveratrol treatment (10–100 μM) reduced Aβ levels by 46% in primary neurons and decreased plaque formation by 54% in the cortex of Tg2576 mice after 45 days of oral administration [126]. Turner et al. (2015) performed a 52-week randomized, placebo-controlled, double-blind trial of resveratrol in 119 patients with mild-to-moderate AD. Participants receiving increasing doses of resveratrol (500–2000 mg/day) showed stabilization of the decline in CSF Aβ42 levels (decreased by only 5.3% versus 29.8% in the placebo group) [127]. Feng et al. (2009) demonstrated that resveratrol treatment (20 mg/kg/day for 8 weeks) in APP/PS1 transgenic mice significantly increased SIRT1 activity, resulting in a 32% reduction in amyloid plaque load and a 38% improvement in spatial learning [128].

### 7.2. Curcumin

Curcumin, the bioactive compound in turmeric, exerts histone acetyltransferase (HAT) and HDAC inhibitory activity and can modulate DNA methylation patterns. Lim et al. (2001) administered dietary curcumin (160–5000 ppm) to aged Tg2576 mice for 6 months, resulting in a 43–50% reduction in insoluble Aβ and a 40% reduction in plaque burden [129]. Findings from the study showed that curcumin treatment inhibited p300 HAT activity by approximately 30%, affecting chromatin remodeling at specific inflammation-related gene promoters [129]. Yang et al. (2022) demonstrated that curcumin restored H3K27 acetylation levels at the BDNF promoter, which had been reduced by 64% following manganese exposure [130]. This epigenetic rescue was accompanied by a 2.1-fold increase in BDNF expression and significant neuroprotection [130]. Pan et al. (2016) demonstrated that 24-week curcumin supplementation (500 mg/kg/day) in 3xTg-AD mice significantly reduced amyloid plaques (68% reduction) and tau aggregates (59% reduction) in the hippocampus and cortex [131].

### 7.3. Epigallocatechin-3-Gallate (EGCG)

EGCG, a catechin abundant in green tea, has shown dual activity in both DNA methylation inhibition and histone modification. EGCG inhibits DNMT1 and reactivates silenced neuroprotective genes. Wang et al. (2022) investigated the effects of EGCG in 6-OHDA-induced PD models both in vitro and in vivo [132]. Treatment with EGCG (25 mg/kg/day for 14 days) in rats significantly protected dopaminergic neurons in the substantia nigra (with 58% more TH-positive neurons compared to the untreated PD model). Molecular analyses revealed that EGCG decreased global DNA methylation by 27% by directly inhibiting DNMT1 activity [132]. Liu et al. (2014) administered EGCG (25 mg/kg/day) to aged rats for 12 weeks and observed significant improvements in spatial memory performance [133]. Their methylation analyses revealed that EGCG treatment reversed age-related hypermethylation at 1342 CpG sites across the genome, while BDNF promoter methylation was reduced by 35% [133]. Singh et al. (2016) demonstrated that EGCG treatment restored the activity of HDAC1 by 52% and HDAC2 by 67%, which had been disrupted by aluminum exposure, with a corresponding 43% decrease in lipid peroxidation [134].

These natural compounds not only offer multi-targeted actions—combining antioxidant, anti-inflammatory, and epigenetic effects—but also possess favorable safety profiles and blood–brain barrier permeability, making them promising candidates for adjunctive therapy in neurodegenerative diseases. However, limitations such as low bioavailability and the need for pharmacokinetic optimization must be addressed in future clinical research.

Incorporating these naturally occurring epigenetic modulators into the therapeutic framework may offer synergistic benefits when used in combination with pharmacological epigenetic agents or as part of nutritional epigenomics strategies. Their inclusion broadens the translational scope of epigenetic therapeutics and reinforces the importance of diet-derived compounds in maintaining cognitive health and preventing neurodegeneration.

## 8. Challenges in Epigenetic Therapeutics

Despite their emerging potential, the clinical application of epigenetic therapies for NDDs faces significant challenges. These obstacles stem from the complexity of targeting epigenetic mechanisms within the CNS while minimizing systemic toxicity and ensuring therapeutic efficacy [135]. Addressing these challenges is critical to realizing the full potential of epigenetic modulators as viable treatments for NDDs.

### 8.1. Tissue-Specific Targeting

One of the primary challenges in epigenetic therapeutics is achieving the brain-specific delivery of drugs [136]. The blood–brain barrier (BBB), a highly selective semi-permeable membrane, limits the entry of many therapeutic agents into the CNS. This barrier protects the brain from harmful substances but also restricts the delivery of potentially beneficial epigenetic drugs, such as HDAC and DNMT inhibitors [137]. As a result, only a fraction of the administered drug reaches the target tissue, reducing its therapeutic efficacy. Off-target effects in peripheral tissues present another significant challenge. Epigenetic drugs often influence gene expression globally, which can lead to unintended modifications in non-neuronal tissues [138]. For example, systemic exposure to DNMT inhibitors can alter DNA methylation patterns in immune or gastrointestinal cells, potentially causing adverse effects. Achieving tissue-specific targeting requires innovative delivery systems, such as nanoparticles, liposomes, or conjugation with CNS-targeting ligands, to enhance drug delivery across the BBB while minimizing systemic exposure [139].

### 8.2. Reversibility of Epigenetic Changes

Epigenetic therapies must achieve reversible and precise modifications to avoid permanent changes that could have unintended consequences [140]. While HDAC and DNMT inhibitors have shown potential in preclinical studies, their long-term effects on epigenetic landscapes remain uncertain [141]. For instance, hyperacetylation or hypomethylation induced by these drugs might lead to the prolonged activation of genes unrelated to the therapeutic target, disrupting normal cellular processes [142]. Moreover, the brain’s highly dynamic epigenetic environment adds complexity to achieving reversibility [143]. Neuronal cells rely on tightly regulated epigenetic mechanisms to respond to environmental stimuli and maintain homeostasis [144]. Broadly altering these mechanisms risks interfering with normal neuronal function. Developing next-generation epigenetic modulators with greater precision and the ability to target specific epigenetic marks or pathways will be essential for overcoming this challenge [145].

### 8.3. Safety and Efficacy

The safety and efficacy of epigenetic modulators remain key concerns, particularly for chronic use in NDDs [146]. The long-term administration of these drugs may lead to off-target effects, such as the dysregulation of non-neuronal genes, which can have systemic consequences [147]. For example, HDAC inhibitors have been associated with gastrointestinal disturbances and immune dysregulation, highlighting the need for more selective compounds [148]. The risk of systemic toxicity is compounded by the immune responses that can arise from global epigenetic alterations. DNMT inhibitors, for instance, may inadvertently activate oncogenes or suppress tumor-suppressor genes, increasing the risk of cancer development [149]. Furthermore, the heterogeneity of NDDs complicates the standardization of treatment protocols. Patient-specific factors, including genetic predispositions and environmental exposures, influence responses to epigenetic therapies, necessitating personalized approaches to ensure efficacy and minimize adverse effects [150].

## 9. Future Directions

The advancement of epigenetic therapeutics for NDDs requires addressing the challenges of delivery, specificity, and variability in patient response [151]. Innovative strategies and emerging technologies are opening new avenues to overcome these obstacles [152].

### 9.1. Development of Brain-Specific Delivery Systems

The effective delivery of epigenetic drugs to the brain remains a critical hurdle due to the restrictive nature of BBB. Nanotechnology-based approaches are being explored to address this limitation. Nanoparticles and liposomes can encapsulate HDAC and DNMT inhibitors, protecting them from degradation in the bloodstream and facilitating their passage across the BBB [153]. These carriers can be engineered to release their payloads specifically in the brain, reducing systemic exposure and off-target effects. Conjugating epigenetic drugs with targeting peptides represents another promising strategy [154]. Peptides designed to bind to receptors expressed on the BBB, such as transferrin or insulin receptors, can ferry drugs into the CNS via receptor-mediated transcytosis [155]. This approach enhances drug specificity and minimizes peripheral toxicity. Additionally, advances in gene therapy techniques, including adeno-associated viral vectors, may provide an alternative for delivering epigenetic modulators directly to affected brain regions [156,157,158,159].

### 9.2. Precision Medicine Approaches

The heterogeneity of NDDs necessitates personalized therapeutic approaches. Precision medicine aims to tailor treatments based on individual genetic, epigenetic, and environmental profiles [160,161,162]. By analyzing a patient’s epigenetic landscape, including DNA methylation patterns and histone modification profiles, clinicians can identify specific dysregulated pathways and select the most appropriate epigenetic modulators [163]. Machine learning and artificial intelligence are expected to play a significant role in precision medicine [164,165,166,167,168]. These technologies can integrate large datasets, including genomic and epigenomic information, to predict patient responses to specific therapies [169]. Precision approaches have the potential to optimize treatment efficacy, minimize adverse effects, and pave the way for personalized interventions that address the unique needs of each patient.

### 9.3. Combination Therapies

Given the multifactorial nature of NDDs, combination therapies are likely to provide the most comprehensive and effective treatment. Merging epigenetic modulators with other neuroprotective agents, such as antioxidants, anti-inflammatory drugs, or mitochondrial enhancers, may offer synergistic benefits [170]. For example, HDAC inhibitors paired with coenzyme Q10, an antioxidant, have shown enhanced neuroprotection in preclinical models of HD. Combination therapies can target multiple pathological pathways simultaneously, addressing both the epigenetic dysregulation and downstream consequences of neuronal damage [171]. However, the careful optimization of dosage and timing is essential to maximize benefits while avoiding interactions or side effects. Future clinical trials should focus on testing these combinations to establish effective protocols for patient care.

### 9.4. Biomarker Development

The development of reliable epigenetic biomarkers is critical for advancing epigenetic therapeutics. Biomarkers can enable early diagnosis, monitor disease progression, and assess the efficacy of treatments [172,173,174,175,176]. Epigenetic changes, such as specific DNA methylation patterns or histone modifications, offer potential as diagnostic and prognostic tools. Emerging technologies like liquid biopsy, which analyzes circulating biomarkers in blood or cerebrospinal fluid, hold promise for low-invasive and highly precise measurements of epigenetic changes. Additionally, integrating epigenetic biomarkers with neuroimaging techniques, such as positron emission tomography (PET) scanning or magnetic resonance imaging (MRI) scanning, may provide a comprehensive view of disease progression and therapeutic response [177]. Standardizing biomarker assays and validating their clinical utility will be essential for their widespread adoption in personalized medicine. Table 3 identifies potential biomarkers, such as global DNA methylation and microRNAs, for monitoring therapeutic efficacy and disease progression, underscoring their importance in personalizing and tracking treatment outcomes [178].

## 10. Limitations of the Review

One major limitation of this review is the lack of systematic quantitative analysis, as no meta-analysis or statistical synthesis of the findings is included across the studies. The conclusions drawn are based on qualitative assessments of the existing literature, which may introduce subjectivity in data interpretation. Additionally, variations in experimental design, sample sizes, and methodologies across preclinical and clinical studies make direct comparisons challenging.

Another limitation is potential publication bias, as studies with positive or significant findings are more likely to be published than those with negative or inconclusive results. This bias may skew the representation of the effectiveness of epigenetic modulators, leading to an overestimation of their therapeutic potential. Moreover, the review relies on publicly available literature from selected databases, which may exclude relevant but unpublished or proprietary research. The heterogeneity of NDDs further complicates the generalization of findings. AD, PD, HD, and ALS have distinct pathophysiological mechanisms, yet they share overlapping epigenetic dysregulation patterns. The extent to which findings from one disease model apply to others remains uncertain, requiring disease-specific validation.

Additionally, clinical translation challenges remain a significant concern. Many promising findings are derived from preclinical studies, which may not fully replicate the complexity of human NDDs. Differences in epigenetic regulation between animal models and humans, as well as the variability in drug response among individuals, limit the direct applicability of preclinical results to clinical settings. Finally, this review does not extensively address long-term safety concerns associated with epigenetic therapies. The potential for unintended gene activation or suppression, immune responses, and systemic toxicity remains an unresolved issue, necessitating further longitudinal studies.

Despite these limitations, this review serves as a valuable resource for the current state of epigenetic therapeutics in NDDs. Future research should prioritize systematic meta-analyses, biomarker-driven patient stratification in clinical trials, and advanced drug delivery technologies to address the challenges highlighted.

## 11. Conclusions

Epigenetic modulators represent a transformative approach to addressing the underlying mechanisms of NDDs. By targeting reversible changes in gene expression, these therapies hold the potential to counteract the epigenetic dysregulation induced by environmental toxins and genetic predispositions. HDAC and DNMT inhibitors have demonstrated efficacy in preclinical models, improving neuronal function and reducing pathological markers. Early clinical trials have also shown encouraging outcomes, particularly with HDAC inhibitors, though the journey to widespread clinical application is fraught with challenges.

Key obstacles include achieving the brain-specific delivery of these agents to overcome the restrictions of the BBB, ensuring that epigenetic changes are precise and reversible, and mitigating the risks of systemic toxicity and off-target effects. The lack of reliable biomarkers available to track therapeutic progress and the variability in patient responses further complicate the translation of these therapies into routine clinical use. Addressing these challenges requires a concerted effort to refine delivery systems, explore long-term safety and efficacy, and leverage advances in personalized medicine to tailor treatments to individual patient profiles.

The future of epigenetic therapeutics lies in innovative strategies such as nanoparticle-based delivery, combination therapies, and the development of robust diagnostic and prognostic biomarkers. With sustained research and technological innovation, these therapies could revolutionize the treatment landscape for NDDs, providing hope for millions of patients worldwide. Continued interdisciplinary collaboration among neuroscientists, clinicians, and bioengineers will be essential to fully realize the potential of epigenetic-based interventions in combating the burden of NDDs.

## Figures and Tables

**Table 1:** Classes of epigenetic modulators and their mechanisms.

Class/Reference	Target	Mechanism of Action	Examples	Application in NDDs
HDAC Inhibitors [25]	Histone deacetylases	Restore histone acetylation; reactivating gene transcription	VPA; Vorinostat	AD, HD
DNMT Inhibitors [25]	DNA methyltransferases	Reverse hypermethylation; restoring gene expression	Azacitidine; Decitabine	PD, HD
BET Inhibitors [26]	Bromodomain proteins	Disrupt the binding of proteins to acetylated histones	JQ1; OTX015	ALS, AD
miRNA Modulators [27]	Non-coding RNAs	Alter microRNA expression to regulate target mRNA levels	Anti-miR-34a	PD, AD
Histone Methylation Modulators [28]	Histone methyltransferases/demethylases	Regulate chromatin states through methylation balance	EZH2 inhibitors	MS, HD

Abbreviations—HDAC: histone deacetylase; DNMT: DNA methyltransferases; BET: bromodomain and extraterminal; MS: multiple sclerosis.

**Table 2:** Emerging drug delivery systems for epigenetic modulators.

Delivery System/Reference	Mechanism	Advantages	Challenges	Examples
Nanoparticles [62]	Encapsulation of drugs	Enhanced BBB penetration; targeted delivery	Limited scalability; potential toxicity	Lipid-based nanoparticles
Liposomes [63]	Lipid bilayer carriers	Biocompatibility; controlled release	Stability issues; production cost	Doxil and epigenetic drug prototypes
Conjugated Peptides [64]	BBB receptor-mediated transport	High specificity; reduced off-target effects	Limited targeting peptides available	Transferrin-conjugated molecules
Viral Vectors [65]	Gene therapy-based delivery	Long-term expression; CNS specificity	Immune responses; insertional mutagenesis	AAV vectors for HDAC inhibitors
Exosome-Based Delivery [66,67,68,69]	Natural vesicle carriers	Biocompatible; minimal immune response	Difficult production; variability	Exosome-encapsulated small RNAs

Abbreviations—BBB: blood–brain barrier; CNS: central nervous system; AAV: adeno-associated virus; HDAC: histone deacetylase.

**Table 3:** Key biomarkers for monitoring epigenetic therapeutics.

Biomarker/Reference	Type	Disease Association	Diagnostic/Prognostic Use	Current Research Status
Global DNA Methylation [179]	Epigenetic modification	AD, PD	Monitor therapeutic efficacy	Preclinical validation
Histone Acetylation [38]	Epigenetic modification	HD	Assess treatment response	Limited clinical application
microRNA-34a [180]	Non-coding RNA	AD, PD	Diagnostic and therapeutic target	Ongoing clinical trials
BDNF Promoter Methylation [181]	Gene-specific DNA methylation	AD	Prognostic indicator	Experimental stage
Circulating Exosomal RNA [182]	RNA encapsulated in exosomes	Various NDDs	Non-invasive monitoring of CNS changes	Emerging research

Abbreviations—BDNF: brain-derived neurotrophic factor; AD: Alzheimer’s disease; PD: Parkinson’s disease; HD: Huntington’s disease; NDDs: neurodegenerative diseases; CNS: central nervous system.
